# Characteristic Features of Childhood and Adolescent Poisonings, in the Mediterranean Region over 6 Years

**Published:** 2018-11

**Authors:** Mustafa KESAPLI, Ahmet CELIK, Ishak ISIK

**Affiliations:** 1. Dept. of Emergency Medicine, Training and Research Hospital, Antalya, Turkey; 2. Dept. of Emergency Medicine, Mehmet Akif Training and Research Hospital, Sanliurfa, Turkey; 3. Dept. of Pediatric Gastroenterology, Training and Research Hospital, Antalya, Turkey

**Keywords:** Poisoning, Childhood, Emergency department, Suicide, Adolescent, Mediterranean

## Abstract

**Background:**

We aimed to define the epidemiological characteristics of poisoning cases in children that have occurred in Antalya, a major city in the Mediterranean.

**Methods:**

The hospital records of children between the ages of 0 to 17 yr admitted to the Pediatric Emergency Department of Antalya Training and Research Hospital, Antalya, Turkey due to poisoning in a 6-year period from 2012–2017 were evaluated.

**Results:**

Overall, 1507 poisoning cases were included in the study, of which 56% were female and 44% were male. Of them, 55% were between the ages of 0 and 5 yr, 10% were between 6 and 12, and 35% were between 13 and 17 year. In the 0–5 yr group, the poisonings were mostly in boys (55.1%) and were all accidental, while in children above 13, the poisonings were mostly in girls (77.8%) and suicide-related (97.1%). The poisonings were due to medication (64.5%) and chemical substances (35.5%). Among medication poisonings, the most common agents were paracetamol (18.0%), NSAID (15.2%) and antibiotics (8.0%). The most frequent chemical substances leading to poisoning were caustic/corrosive chemicals (22.8%) pesticides (8.0%). Interventions most commonly administered were activated charcoal (60.9%), gastric lavage (38.6%) and naso-gastric catheter (36.6%). Mortality was observed in 2 cases during six years.

**Conclusion:**

Knowledge on epidemiological and clinical features of poisoning in children according to age groups, establishing drug and chemical substance safety for children, and widespread parent education shall help decreasing childhood poisoning.

## Introduction

Poisoning is defined as a clinical condition that develops after an external toxic substance leads to damage or death in cells. Poisoning may occur by ways of oral ingestion, inhalation, injection and absorption. The severity of poisoning varies according to the type, dosage, formulation and mode of transmission of the chemical, the age and nutritional condition of the person affected, existence of the other diseases, and other factors (1).

Poisoning due to medication and chemical substances is a major cause of mortality and morbidity in children. While poisoning occurs in every age group, it is more frequent and more likely to lead to death in childhood (2).

Approximately 56% of poisonings in Turkey occur in children according to a study conducted at the Refik Saydam Hygiene Institute (3). 64.5% of the poisonings occur in persons under the age of 19, according to 2006 data of the American Association of Poison Control Centers (4).

Poisoning remain as an important death risk in childhood even after increased socioeconomic levels of societies, decreased rates of infectious diseases and diseases related to malnutrition, and improved treatment procedures (5). The childhood age group constitutes approximately two thirds of poisoning cases and children under the age of 5 constitute 80% of them (6). The incidence of medication poisoning in children varies between 0.33% and 7.6% (7).

While the cause of poisoning in the preschool age group is generally medication found at home and unintentional intake of the caustic/corrosive cleaning products, the reason is mostly suicide attempts in the school and adolescence period (8). Public education and proper storage of medicine and chemical substances at home play a key role in preventing such poisonings (9).

The epidemiological features of poisoning, which is a global problem, vary according to socioeconomic regions. In order to decrease the mortality and morbidity rates due to poisoning, knowledge of the regional factors is crucial (10).

The purpose of this descriptive study was to make a contribution to the development of measures to decrease mortality and morbidity by determining the epidemiological features of the cases admitted due to poisoning in children.

## Materials and Methods

The hospital records of the cases admitted to the Emergency Department of Antalya Training and Research Hospital, Antalya, Turkey between 01.01.2012 and 31.12.2017 with a diagnosis of poisoning have been retrospectively investigated according to the International Classification of Diseases-Code 10. The patients were separated in three age groups of 0 to 5, 6 to 12 and 13 to 17. Food, mushroom and plant poisonings were not included in the study.

The poisoning cases were classified with the following parameters: age, gender, single or multiple substance intake, physical examination and symptoms, active chemical taken, purpose of intake, arrival time to the emergency department, treatment methods administered, treatment period, and treatment results.

An approval was obtained from the Ethics Committee of Noninvasive Research of Antalya Training and Research Hospital.

The SPSS for Windows ver. 20.0 (Chicago, IL, USA) program was used for the statistical analyses while determining the findings. The frequency distributions, averages and standard deviations in the study were determined.

## Results

Overall, 1507 cases including 851 girls (56.5%) and 656 boys (43.5%) were admitted to the Pediatric Emergency Department due to poisoning. Ages 0–5 preschool period group constituted 830 (55.0%) of all cases, where boys were more prevalent with 458 cases (55.1%). In the ages 13–17 yr adolescence group, girls constituted the majority with 411 cases (77.8%). The boy/girl ratio was 1.3/1 throughout the study group. The average age was 7.43 overall, 9.10 for girls and 5.26 for boys.

The majority of the poisoning cases in the pre-school age group were accidental (830 cases; 83.6%), while most of the cases involving adolescent children aged 13–17 were suicide-related (500 cases; 97.0%) (Table 1).

**Table 1:** Causes of poisoning according to age groups

***Cause***	***0–5 age n (%)***	***6–12 age n (%)***	***13–17 age n (%)***	***Total n (%)***
Accident	830 (83.6)	134 (13.5)	28 (2.8)	992 (65.8)
Suicide	-	15 (2.9)	500 (97.0)	515 (34.1)
Total	830 (55.0)	149 (9.8)	528 (35)	1507 (100.0)

Among this adolescent group attempting suicide, girls were more prevalent with 411 cases (77.8%). The most frequent medication leading to poisoning in all age groups were specified as paracetamol (18.0%), NSAID (15.2%) and antibiotics (8.0%) (Table 2). In the age group of 0–5 years, chemical substances were more frequently the cause of poisoning (24.8%), compared to medication. Among these substances were caustic/corrosive agents used in cleaning (73.9%), pesticides (11.2%), mouse poison (5.8%) and naphthalene (2.9%). In the adolescent group (ages 13–17), pesticides (73.9%) and caustic/corrosive substances (20.6%) were the most prevalent poisoning agents (Table 3).

**Table 2:** Medications leading to poisoning according to age groups

***Medication***	***0–5***		***6–12***		***13–17***		***Total***	
	***n***	***%***	***n***	***%***	***n***	***%***	***n***	***%***
Paracetamol	88	17.5	10	11.7	174	19.9	272	18.0
NSAID	72	14.3	10	11.7	148	16.9	230	15.2
Antibiotics	43	8.5	6	7.0	72	8.2	121	8.0
SSRI	17	3.3	5	5.8	82	9.4	104	6.9
Pseudoephedrine	24	4.7	4	4.7	73	8.3	101	6.7
Antipsychotic	17	3.3	7	8.2	53	6.0	77	5.1
Vitamins and minerals	27	5.3	6	7.0	32	3.6	65	4.3
GIS drugs ı (antiemetic, antispasmodic, anti- hemorrhoid)	19	3.7	6	7.0	40	4.8	65	4.3
Antihypertensive	33	6.5	4	4.7	25	2.8	62	4.1
Antihistaminic	12	2.3	4	4.7	45	5.1	61	4.0
SSS drugs I (antiparkinson, antivertigo)	19	3.7	6	7.0	21	2.4	46	3.0
Respiratory system mucolytic, antitussive)	25	4.9	3	3.5	14	1.6	42	2.7
TCA	12	2.3	4	4.7	20	2.2	36	2.3
Antiaritmic	16	3.1	0	0.0	17	1.9	33	2.1
Myorelaxant	7	1.3	1	1.1	20	2.2	28	1.8
Endocrine system (antityroid, antilipid)	20	3.9	1	1.1	5	0.5	26	1.7
Oral contraseptives	12	2.3	4	4.7	5	0.5	21	1.3
Benzodiazepine	9	1.7	1	1.1	11	1.2	21	1.3
Antineoplastic	5	0.9	1	1.1	2	0.2	8	0.5
Other	14	2.7	1	1.1	12	1.3	27	1.8

NSAID: Non-Steroid Anti Inflammatory Drugs, SSRI: Selective Serotonine Re-uptake Inhibitor, TCA: Tricyclic Antidepressant

**Table 3:** Types of chemical substances leading to poisoning according to age groups

***Substance***	***0–5***		***6–12***		***13–17***		***Total***	
	***n***	***%***	***n***	***%***	***n***	***%***	***n***	***%***
Caustic/corrosive agents	276	73.9	50	68.4	19	20.6	345	22.8
Pesticide	42	11.2	12	16.4	68	73.9	122	8.0
Mouse poison	22	5.8	4	5.4	3	3.2	29	1.9
Naphthalene	11	2.9	3	4.1	0	0	14	0.9
Cigarette	8	2.1	0	0	0	0	8	0.5
Ethyl alcohol	5	1.3	1	1.3	0	0	8	0.5
Plant based	7	1.8	3	4.1	0	0	10	0.6
Methanol	2	0.5	0	0	0	0	2	0.1
Ethylene glycole	1	0.2	0	0	0	0	1	0.06
Total	373	69.3	73	13.5	92	17.1	538	35.36

Majority of cases (66.6%) had no complaint at the time of admission to the emergency department.

The most frequent complaints specified in all age groups were nausea-vomiting (14.9%), state of sleep (5.3%) and abdominal pain (4.8%).

Generally, no pathological signs were detected by physical examination in all age groups (87.3%). Confusion (4.9%), epigastric sensitivity (3.1%) and hyperemia in the oral mucosa (2.1%) were determined as the most frequent findings during physical examination. 79.5% of the cases were reported to be caused by a single agent, 10.1% by two agents, 5.9% by three agents and 2.8% by four agents. One case was due to eight agents and another one involved twelve agents. The period of time that passed between intake and admission to the emergence department was as follows: 254 cases (16.8%) were admitted in the first 30 minutes, 767 cases (50.9%) in the first 1 to 2 hours and 217 cases (14.4%) in 2 to 3 hours (Table 4).

**Table 4:** The period of time between intake and admission according to age groups

***Period***	***0–5***		***6–12***		***13–17***		***Total***	
	***n***	***%***	***n***	***%***	***n***	***%***	***n***	***%***
Not known	3	0.2	1	0.1	4	0.3	7	0.6
First 30 minutes	170	11.3	24	1.6	60	4.0	254	16.8
1 – 2 Hours	462	30.7	70	4.6	235	15.6	767	50.9
2 – 3 Hours	103	6.8	30	2.0	84	5.6	217	14.4
3 – 6 Hours	65	4.3	17	1.1	71	4.7	153	10.1
6 – 12 Hours	21	1.4	4	0.3	44	2.9	69	4.6
12 – 24 Hours	2	0.1	1	0.1	22	1.5	25	1.7
24 – 48 Hours	3	0.2	1	0.1	5	0.3	9	0.6
48 – 72 Hours	2	0.1	1	0.1	2	0.1	5	0.3

The most frequent treatment interventions were activated charcoal (60.9%), gastric lavage (38.6%), naso-gastric catheter (36.6%), non-invasive monitoring (36.5%), serial ECG follow-up (13.2%), and foley catheter (5.8%) (Table 5). The number of cases where antidotes was used were as follows: nalaxone in 2 cases, atropine in 2 cases, atropine+PAM in 3 cases, fomepizole in 6 cases and vitamin K in 1 case.

**Table 5:** Types of treatment interventions according to age groups

***Treatment***	***0–5***		***6–12***		***13–17***		***Total***	
	***n***	***%***	***n***	***%***	***n***	***%***	***n***	***%***
Activated charcoal	422	28.0	61	4.0	435	28.9	918	60.9
Gastric lavage	277	18.4	25	1.7	279	18.5	581	38.6
Naso-gastric catheter	259	17.2	21	1.4	271	18.0	551	36.6
Non-invasive monitoring	227	15.1	39	2.6	283	18.8	549	36.5
Serial ECG follow-up	62	4.1	12	0.8	125	8.3	199	13.2
Foley catheter	18	1.2	1	0.1	68	4.5	87	5.8
Blood-sugar follow-up	11	0.7	0	0.0	28	1.9	39	2.6
Invasive monitoring	1	0.1	0	0.0	6	0.4	7	0.5
Endotracheal intubation	1	0.1	0	0.0	5	0.3	6	0.4
Arterial line	1	0.1	0	0.0	5	0.3	6	0.4
Central catheter	0	0.0	0	0.0	4	0.3	4	0.3
Cardiac pacemaker	0	0.0	0	0.0	1	0.1	1	0.1
CPR	0	0.0	0	0.0	2	0.1	2	0.1

After initial observation in the emergency department, 93 patients (6.2%) were discharged, 947 patients (62.8%) were admitted as inpatients and 430 patients (27.9%) were placed in the intensive care unit. Additionally, 47 patients (3.1%) left the hospital by their families’ will.

99.8% of the cases were treated without any sequela. Two cases (0.1%) in the age group of 13–17 years resulted in death. One case (0.06%) developed related sequela.

## Discussion

Overall, 1507 of poisoning cases in children aged 0 to 17 over a 6-year period were investigated in our study. Of the study group, 44% were boys and 56% were girls. A similar study included a study group of 55% girls (10). The number of boys and girls were equal in another study conducted in a region close to ours (11).

When the poisoning cases were divided according to age groups, the prevalence of boys in the ages 0–5 preschool period (55%) and that of girls in the ages 13–17 adolescence period (77.8%) drew attention. This showed that boys in early ages and girls in the adolescent period were subjected to poisoning more often. In a 33-year survey, the poisoning cases peaked in boys under the age of 5 and in girls over the age of 13 (12). In France girls suffered poisoning most frequently in the age range of 12 to 15 (13).

In our study, the majority of poisoning cases involved children in the preschool age group (55%). Similar rates have been reported in comparable studies in the literature. Fifty one percent of childhood poisonings happened to children under the age 6, while 38% of these were 3 years old or younger (14). In a study, 67% of poisonings involved children under the age of 4 (15). Besides, 58% of children subjected to poisoning were younger than 5 years old (10). In this developmental stage, children use all their senses in order to discover their surrounding environment. The sense of taste is one of them. Children can easily reach medication and household chemicals that are not kept safely, and may be subjected to poisoning (1).

In our study, accidental poisoning cases occurred mostly in the preschool age group (65.8%), while suicide-related poisonings were more frequent in the adolescence period (34.1%). In the USA, the suicidal thoughts and attempts are among the prevalent mental health emergencies in the adolescent period (16), and suicide is the 3^rd^ leading cause of death in the age range of 10 to 24 (17). Poisonings in children aged 0–5 occurred mostly as a result of accidents (18).

As for poisoning agents, another study showed that the most prevalent causes of medication poisoning were analgesic and antipyretic agents, the most common one being paracetamol (19). Medication had started coming to the forefront in non-fatal poisoning cases in countries with medium and high income. In United Arab Emirates, in children aged 1 to 5 yr, analgesics, NSAID, and antihistaminics were the most common cause of poisoning (20). Children are poisoned with medication that they can reach easily, which are also what they and their parents use most often. Therefore, improving medication packaging and safety measures is of importance (21).

In our study, we determined that in the ages 13–17 yr group, the most prevalent medications involved in poisoning were paracetamol, NSAID, antibiotics, SSRI, pseudoephedrine and antipsychotics. Ramisetty et al. reported that among 1246 patients aged 10–17 that suffered poisoning, 75% were girls and 65% were between the ages of 15 and 17 (21). In the same study, the most common agents were paracetamol (53%) and psychotropic agents (21%). Chafe et al. identified the mean age of adolescent poisoning as 15.4 in a study group of mostly girls (78.2%), in which analgesics (38.0%) and antidepressants (32.2%) were the most ingested medications. Also, they detected that admissions for poisoning increased over the study phase from 2.1% of total hospital admissions in 2008 to 6.5% in 2013 (22). Suicide rates among young people are high, especially among girls (23). In children aged 12–16, sedative-hypnotics (33.1%), psychotropics (27.3%) and analgesics (17.6%) were used the most (23).

The higher prevalence of girls in poisoning cases might be due to generally earlier mental and physical maturation of females and thus encountering emotional and behavioral problems earlier in life compared to males (24).

Our study identified the most frequent nonpharmacological substances involved in poisoning as pesticides (73.9%) and caustic/corrosive agents (20.6%). Around Zagazig, which is an agricultural region in Egypt, pesticides were the most common poisoning substances (25). Antalya is one of the most important regions where agricultural crops are grown in Turkey. The reason why pesticides are often involved in poisoning might be that commercial agriculture is widespread and these chemicals can be obtained with ease in an uncontrolled manner.

In our study, the proportion of poisoning cases involving chemical substances in the preschool period (ages 0–5) was 24.8%. These substances were caustic/corrosive substances used in house cleaning (18.3%), pesticides (2.8%) and mouse poison (1.5%). 44.3% of the total cases of poisoning were with corrosive substances (12). Accidental poisoning is especially common in children in play age, since they are hyperactive and can confuse cleaning substances with food. Throughout our study group, 79.5% of poisonings were with a single drug, 10.1% with two drugs and 5.9% with three drugs. The proportion of poisoning cases involving only one agent was 91% (26). Similarly, in Turkey, 81.3% of poisonings were due to exposure to a single agent. The reason behind the multiple drug intakes seen in the adolescent group is probably that the poisonings resulted from suicide attempts in this group (12).

Most of the children (88.7%) had no complaints when they arrived at the hospital. Gastrointestinal system complaints (nausea, vomiting, abdominal pain) were the most frequent complaints (6.7%). 71.7% of the cases were symptomatic at the time of admission to the hospital and that the most common complaints were nausea-vomiting (37.5%) and fever (20.0%) (27). In addition, 46% of patients had no complaints, while the most frequent complaints were nausea-vomiting (22%) (28).

On physical examination, no pathological findings were determined in 87.3% of our study group, while confusion (4.9%) and hyperemia in the oral mucosa (2.1%) were the most common complaints. Confusion was frequently observed in the 13–17 years of age group, which involved the highest rate of suicide-related poisoning. This condition is likely to be due to intake of SSRIs and antipsychotic drugs. In children aged 0 to 5, hyperemia in the oral mucosa (1.7%) were especially specified and is considered to be depending on the frequent caustic/corrosive substance in-take in this age group. Salvatore et al. reported 18.8% of hyperemia during physical examination (28). In paediatric emergency departments, when a child has nonspecific physical examination findings, poisoning should be considered as differential diagnosis.

In our study, 16.8% of cases arrived at the emergency department in the first 30 minutes, 50.9% within two hours and 92.2% in the first 6 hours. 80.8% of poisoning cases were admitted to the emergency department within 4 hours (27). Similarly, most cases arrived at the emergency department in the first 6 hours (12). Accordingly, it could be stated that families are sensitive to poisoning and try to arrive at the emergency department as early as possible.

As for administered treatment interventions in our study, 50.9% of the patients applied in 1 to 2 hours suggested for gastric lavage and activated charcoal applications. Therefore, interventions of activated charcoal and naso-gastric catheter were administered more often than recommended by guidelines that specify selected cases (according to the characteristics of the substance and the period of time since intake). The reason for this might be that the specialists working in the emergency department use these interventions more frequently in order to protect themselves against legal ramifications. Ghaffar et al. applied gastric lavage on 35% of the cases (27). In Norway, naso-gastric lavage was used at the rate of 36% in the 1980s, and that rate regressed to 9% in 2003–2005 (29).

In our study group, 6.2% of patients were discharged after the observation period in the emergency department, 62.8% were admitted to the paediatrics department and 27.9% were admitted to the intensive care unit. A small fraction of children (3.1%) left the hospital by their families’ will. Of the observed inpatients, 55.7% were discharged 24 hours later, 22.0% after 48 hours and 5.0% after 72 hours (5%). Overall, 82.7% of the patients were discharged within the first 72 hours. About, 97% of poisoning cases were discharged following a 3-day follow-up period (30).

## Conclusion

Especially in the preschool ages, children have an elevated curiosity, move on the floor and come across a plethora of foreign objects that they are keen on orally investigating, which leads to accidental poisoning. The poisoning agents are medication taken by the children and their parents, and caustic/corrosive substances used at home. In order to prevent childhood poisoning, children should be provided with safer living conditions, medication and chemical substances should be kept in secure packages at unreachable places, child-proof bottle caps should be more widespread, and public education should be made available to more people. Suicide-related poisonings especially in the adolescent age group should be attentively followed-up with school and family cooperation. Also in this age group, psychologically supportive interventions must be provided regularly.

## Ethical considerations

Ethical issues (Including plagiarism, informed consent, misconduct, data fabrication and/or falsification, double publication and/or submission, redundancy, etc.) have been completely observed by the authors.
